# Yessotoxin, a Marine Toxin, Exhibits Anti-Allergic and Anti-Tumoural Activities Inhibiting Melanoma Tumour Growth in a Preclinical Model

**DOI:** 10.1371/journal.pone.0167572

**Published:** 2016-12-14

**Authors:** Araceli Tobío, Amparo Alfonso, Iris Madera-Salcedo, Luis M. Botana, Ulrich Blank

**Affiliations:** 1 INSERM UMRS 1149, Paris, France; 2 Departamento de Farmacología, Facultad de Veterinaria, Universidad de Santiago de Compostela, Campus Universitario, Lugo, Spain; 3 CNRS, Center de Recherche sur l’Inflammation, Equipe de Recherche Labellisée (ERL), Paris, France; 4 Université Paris Diderot, Sorbonne Paris Cité, Laboratoire d’excellence INFLAMEX, Paris, France; IDI, Istituto Dermopatico dell'Immacolata, ITALY

## Abstract

Yessotoxins (YTXs) are a group of marine toxins produced by the dinoflagellates *Protoceratium reticulatum*, *Lingulodinium polyedrum* and *Gonyaulax spinifera*. They may have medical interest due to their potential role as anti-allergic but also anti-cancer compounds. However, their biological activities remain poorly characterized. Here, we show that the small molecular compound YTX causes a slight but significant reduction of the ability of mast cells to degranulate. Strikingly, further examination revealed that YTX had a marked and selective cytotoxicity for the RBL-2H3 mast cell line inducing apoptosis, while primary bone marrow derived mast cells were highly resistant. In addition, YTX exhibited strong cytotoxicity against the human B-chronic lymphocytic leukaemia cell line MEC1 and the murine melanoma cell line B16F10. To analyse the potential role of YTX as an anti-cancer drug *in vivo* we used the well-established B16F10 melanoma preclinical mouse model. Our results demonstrate that a few local application of YTX around established tumours dramatically diminished tumour growth in the absence of any significant toxicity as determined by the absence of weight loss and haematological alterations. Our data support that YTX may have a minor role as an anti-allergic drug, but reveals an important potential for its use as an anti-cancer drug.

## Introduction

Yessotoxins (YTXs) are a group of lipophilic marine exotoxins produced by the dinoflagellates *Protoceratium reticulatum*, *Lingulodinium polyedrum* and *Gonyaulax spinifera* [1–3]. The group of YTXs is composed of close to 90 known analogs (YTX, homoYTX, hydroxyYTX, carboxyYTX and desulfoYTX among others) [4], however, the chemical structures for most of these compounds remain unclear [1]. Although no lethality was observed after oral administration in mice (doses up to 54mg/Kg), YTXs were reported to be lethal after intraperitoneal injection causing restlessness, dyspnoea, shivering, jumping and cramps albeit at relatively high levels of median lethal dose (LD_50_) values ranging from 80 to 750μg/Kg depending on the mouse strain used [5]. YTX is the most toxic among all the analogous, nevertheless, the toxicity following intraperitoneal injection or oral administration at sublethal doses does not induce neither macroscopic nor histological alterations, even in the cardiac muscle [6, 7]. Another study, however, reports some moderate changes and damage in the myocardium that are reversible in the long-term [8, 9].

The role of YTX compounds as marine seafood contaminant toxins is presently debated. Based on the toxicity observed in mouse bioassays and their coexistence with other marine toxins (okadaic acid and dinophysistoxins) they have been included in the list of marine toxins [8]. While the European Food Safety Authority (EFSA) sets an Acute Reference Dose (ARfD) of 25μg YTX equivalents/Kg body weight, the European Union has established a limit of 3.75mg YTX/Kg of shellfish meat, as a prevention measure [10]. Yet, it remains a fact that the biological activity of YTXs in the environment is incompletely understood.

In order to better understand the mechanism of action of YTXs some of their activities have been studied *in vitro* in previous works. An intriguing finding revealed a cytotoxic effect of YTX described initially in rat glioma cells [11] and hepatocytes [12]. Subsequent studies performed in BE(2)-M17 neuroblastoma demonstrated that YTX induces apoptotic cell death [13]. Likewise, tumoural K-562 lymphocytes were shown to be YTX-sensitive dying by apoptosis, while normal human lymphocytes proved to be YTX-resistant [14, 15]. In some mouse cell lines autophagy activity was found after YTX exposure [16]. However, no *in vivo* studies testing the cytotoxic effects of YTX in the treatment of tumours have been reported so far.

In addition to cytotoxicity, anti-asthmatic and anti-allergic effects have been proposed for the YTX group, albeit these therapeutic effects are not completely understood [17, 18]. The interaction of allergens and allergen-specific IgE with the high-affinity IgE receptor (FcεRI) represents the key event in type I hypersensivity allergic reactions triggering a signalling cascade enabling rapid release of multiple inflammatory mediators such as histamine from mast cells (MCs) and basophils [19]. MCs are derived from CD34^+^/c-kit^+^ progenitor cells and play a crucial role in the pathogenesis of allergy, inflammation and many chronic inflammatory processes [20]. However, no studies examining directly the effect of YTX on the viability and cellular activation as well as the degranulation of MCs have been reported so far. We therefore sought to examine the activities of YTX on MC biology. Our results show that the anti-allergic effect is minor, while the toxin profoundly inhibits the growth of tumour MCs and some other tumour cells, which undergo apoptosis, while primary bone marrow derived MCs were YTX resistant. We also studied the cytotoxic properties of YTX *in vivo* using the well-established B16F10 melanoma model in mice to clarify the capacity of this molecule as an anti-cancer compound.

## Materials and Methods

### Reagents

YTX (purity ≥ 99%) dissolved in methanol was obtained from Cifga Laboratorio (Lugo, Spain). 4-Nitrophenyl N-acetyl-β-D-glucosaminide (pNAG), propidium iodide solution, MgCl_2_, glucose, DNP-human serum albumin (DNP-HSA) and 3-[4,5-dimethylthiazol-2-yl]-2,5-diphenyltetrazolium bromide (MTT) were from Sigma-Aldrich (Germany). Annexin V-FITC and 10X AnnexinV Binding Buffer were from BD Pharmingen (France). HEPES was from Gibco (Life Technologies). NaCl was from VWR (Belgium). KCl and Triton X-100 were from Euromedex (France). CaCl_2_ was from Prolano (Paris, France). Bovine serum albumin (BSA) was from PAA (Austria). Mouse monoclonal anti-DNP IgE has been described previously [21–23].

### Animals

The C57/BL6 strain was raised and maintained at the animal facilities of the Center of Research on Inflammation. All experiments were performed in accordance with the national ethical guidelines and with the approval of local authorities of the Comité d’Éthique Expérimentation Animale Bichat-Debré. Animals were in cages with the following dimensions: 30x20x13 (LxWxH) and separated by three groups (Untreated, Vehicle and Yessotoxin group). Each cage was labeled with a card indicating the study, group and animal number. A maximum of 3 animals of the same group were housed in the same cage. Mice were maintained in a specific pathogen-free facility with a 12-h light/12-h dark cycle. Sterile food and sterilized pure water was provided ad libitum during the whole procedure.

### Cells and cell lines

Bone marrow-derived mast cells (BMMCs) were derived from the bone marrow of C57BL/6 mice. The mice were sacrificed by CO_2_ inhalation and bone marrow cells were cultured in IMDM containing 15% fetal bovine serum (FBS) (Gibco, Life Technologies), 1mM sodium pyruvate (GE Healthcare), 100μM non-essential amino acids (Gibco, Life Technologies), 100UI/mL penicillin, 100μg/mL streptomycin (Gibco, Life Technologies) and 0.05mM 2-mercaptoethanol ≥ 99% (Gibco, Life Technologies). The medium is supplemented with 10ng/mL interleukin-3 and 10ng/mL Stem Cell Factor (both from Miltenylbiotec). Cells were cultured in an atmosphere containing 5% CO_2_. After five weeks in culture more than 95% of the cells were MCs assessed by toluidine blue staining.

RBL-2H3 and B16F10 melanoma (kindly provided by Dr. Thiago Maciel, Imagine Institute, Paris, France) cell lines were grown at 37°C in DMEM-Glutamax High Glucose medium (Gibco, Life Technologies) containing 10% FBS, 100UI/mL penicillin, 100μg/mL streptomycin (Gibco, Life Technologies) and for B16F10 additionally 1mM sodium pyruvate (Gibco, Life Technologies), 100μM non-essential amino acids and 0.05mM 2-mercaptoethanol ≥ 99% (Gibco, Life Technologies) in an atmosphere containing 5% CO_2_. The MEC1 cell line (kindly provided by Dr. Pierre Launay, INSERM U1149, Paris, France) was maintained at 37°C in RPMI-Glutamax medium (Gibco, Life Technologies) supplemented with 10% FBS, 100UI/mL penicillin and 100μg/mL streptomycin (Gibco, Life Technologies) in an atmosphere containing 5% CO_2_.

### β-hexosaminidase release measurement

Cells (2x10^6^/mL) were incubated with monoclonal anti-DNP IgE ascites fluid (1:10,000 final dilution) overnight. After washing, cells were resuspended in 500μL Tyrode's buffer (20mM HEPES pH 7.2, 137mM NaCl, 5mM KCl, 1mM MgCl_2_, 1,8mM CaCl_2_, 5,6mM glucose, 0,5mg/mL BSA). Cells were then treated with indicated concentrations of YTX or vehicle (methanol) for 30 min and 1h before stimulation for 45min with DNP-HSA in the presence or absence of YTX as indicated. The reaction is stopped by immersing cells on ice for 10min. The percent released β-hexosaminidase into the supernatant was then determined as previously described [24], after subtracting background release of non-stimulated cells. 100% values were determined after addition of 0.5% Triton X-100 to lyse the cells.

### Determination of cellular viability and apoptosis/necrosis

Cell viability was measured using the MTT test. This assay measures mitochondrial function by determining the quantity of formazan formed after conversion of the soluble MTT dye by active mitochondrial dehydrogenases in live cells. Briefly, after incubation with YTX cells (0.5x10^6^/mL) were washed in 500μL Tyrode's buffer before addition to MTT (250μg/mL) and incubation for 30min at 37°C [14, 25, 26]. After incubation cells were centrifuged and resuspended in 100μL H_2_O and sonicated. Final optical density obtained from formazan formation was measured at 595nm with a multi-mode plate reader (Infinite M200, Tecan^®^). Cells lysed in 10% Triton X-100 were used as 100% positive control for death cells.

Apoptosis and necrotic cell death were determined by flow cytometry using AnnexinV-FITC/propidium iodide (PI) staining. Cells (1x10^6^cells/mL) were incubated with different concentrations of YTX for indicated times. Cells were washed with Tyrode's buffer, resuspended in 100μL 1X AnnexinV Binding Buffer and stained by adding 5μL of AnnexinV-FITC (BD Pharmingen) for 15min at RT as well as of 2.5μg/mL PI solution (Sigma-Aldrich). Flow cytometry data were acquired on a Fortessa flow cytometer (BD Biosciences) and analysed using FlowJo software (TreeStar). Live cells were considered both Annexin V-FITC and PI negative; early apoptotic cells were considered Annexin V-FITC positive and PI negative, late apoptotic cells were considered both Annexin V-FITC and PI positive and necrotic cells were considered Annexin V-FITC negative and PI positive.

### B16F10 mouse model of subcutaneous melanoma

Anti-cancer effect of YTX *in vivo* was studied using the B16F10 melanoma mouse model. A total of 2x10^5^ B16F10 melanoma cells in 100μL PBS were injected subcutaneously into the flank of C57BL/6 females (7 weeks) inducing within 5 to 10 days a palpable tumour of about 50mm^3^ [27]. At this time point, mice were treated with 100μg/Kg of YTX or vehicle administrated subcutaneously in close vicinity to the site of tumour development. Additional lower doses of YTX (20μg/Kg and 10μg/Kg) were administrated subcutaneously as indicated. As the B16 melanoma is a fast growing tumour, its volume was determined daily until sacrifice by the following formula for solid tumours: volume = (length x width^2^)/2 [28, 29]. Treatment time was kept to a minimum of 5 days and mice were euthanized by CO_2_ inhalation (5 L/min) for 10 minutes at day 12 allowing a maximum of 7 days of tumour growth before they reached necrotic or ulcerating stages as previously recommended [27]. Signs like no interest on cage exploration, weight and/or appetite loss, difficulty in breathing and loss of coordination were evaluated two times per day. Additional signs of toxicity such us difficulty to eat, drink, walk or groom, distress or moribund signs or any other signs of systemic toxicity were also monitored for immediate euthanization. None of the animals suffered from any of these signs during the procedure, therefore, no animals were euthanized during the study. The maximal tumour volume achieved was in the range or lower of previously published data [30–34]. The weight loss at the day of sacrifice considering tumour density [35] was less than 20%. A blood sample was collected before sacrifice and immediately analysed in the Melet Schloesing Haematology Analyser for haematological parameters determination including lymphocytes, monocytes, neutrophils/granulocytes, erythrocytes and platelets. All experiments were performed in accordance with the national ethical guidelines and with the approval of local authorities of the Comité d’Éthique Expérimentation Animale Bichat-Debré.

### Glucose uptake measurement in B16F10 mouse model of subcutaneous melanoma

At day of sacrifice 100μL of RediJect 2-DG Fluorescent Imaging Probe (Perkin Elmer^®^) were injected intravenously to visualize glucose uptake known to be directly related to cell proliferation and hence tumour growth. After 3h, animals were anesthesized with 5mg Ketamine (Ketamine 50mg, Virbac^®^) and 20mg Xilacin (Rompun 2%, Bayern^®^) diluted in PBS and injected intraperitoneally. Luminiscence images were captured using a FX Pro (Kodak) and analyzed with Carestream MI software.

### Statistical analysis

One-way ANOVA was employed for comparison of significant differences among groups. A probability level of 0.05 or smaller was used for statistical significance.

## Results

**Fig 1:**
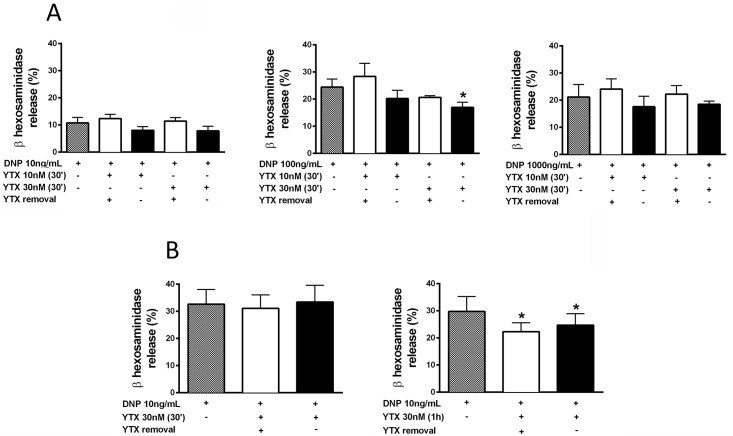
Effect of YTX on β-hexosaminidase release induced by IgE/Ag in RBL-2H3 and BMMCs. IgE-sensitized RBL-2H3 cells (A) or BMMCs (B) were incubated with 10 and 30nM YTX or vehicle (methanol) for 30 or 60min. Then YTX was removed (+) from the medium or not (-) and cells were stimulated for 45min with DNP-HSA antigen at the indicated concentrations. The percentage release of β-hexosaminidase was determined and compared to vehicle treated cells. No effect was observed after vehicle incubation. Data (percentage release) are the mean ± SEM of three experiments. Significant differences between DNP-HSA- (hatched) and DNP-HSA+YTX-treated cells: (*) p≤0.05.

To analyse whether the reported anti-allergic and anti-asthmatic activity of YTX involved its ability to inhibit MC degranulation we evaluated the effect of YTX on the release of granule-stored β-hexosaminidase in the RBL-2H3 MC line and in primary BMMCs. IgE-sensitized RBL-2H3 cells and BMMCs were treated with 10 or 30 nM YTX for 30 or 60min before stimulating them with specific antigen (DNP-HSA) for 45min in the presence or absence (removal) of YTX. In RBL-2H3 cells no significant effect was observed at low and high antigen doses (10ng/mL and 1000ng/mL) while at more optimal stimulatory doses (100ng/mL), a slight decrease in the degranulation response was observed at 30nM of YTX, particularly when the toxin remained present during the stimulation period (Fig 1A). A decrease of the degranulation response was also observed in BMMCs after 1h incubation of YTX, independently of whether it had been removed before stimulation or not (Fig 1B, right panel). The inhibitory effect required prolonged preincubation with YTX as it was only observed after pretreatment for 1h, while no effect was observed after 30min (Fig 1B, left panel). Although these results demonstrate that YTX decreases β-hexosaminidase release, its effect was not very potent reaching with a maximum inhibition of 25% in both BMMCs and RBL cells. It also appeared to be dependent on the dose of antigen in RBL-2H3 cells disappearing at non optimal stimulatory doses (Fig 1A).

**Fig 2:**
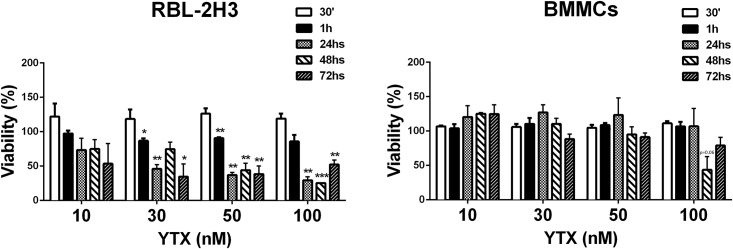
Effect of YTX on RBL-2H3 and BMMCs cell viability. Cells were incubated with 10, 30, 50 and 100nM YTX for the indicated times and cell viability was assessed by the MTT assay. Corresponding controls with YTX solvent (methanol) were performed and cell viability was arbitrarily set to 100%. Of note, solvent did not significantly affect cell viability as compared to non-treated cells even at the highest concentration of vehicle. Data are the mean ± SEM of three experiments. Significant differences between untreated and YTX-treated cells: (*) p≤0.05, (**) p≤0.01 and (***) p≤0.001.

As YTX was shown previously to be toxic to tumour cells at relatively low concentrations, we performed control experiments to evaluate whether the toxin had any effect on MC viability. Fig 2 shows that using the MTT assay YTX did not induce any cytotoxic effect neither in BMMCs nor in RBL-2H3 after short (30min) incubation times. However, after 1h of incubation time a slight but significant decrease or tendency to decrease in cell viability was observed in RBL-2H3 cells at the high YTX concentrations (30, 50 and 100nM). This effect became highly prominent after 24, 48 and 72h suggesting that the toxin exhibited significant toxicity towards the RBL-2H3 MC cells line. Surprisingly, the effect was different when BMMCs were examined as no significant toxicity of YTX could be observed. Only with the highest concentration of YTX (100nM) a tendency for some toxicity was observed at 48h and 72h of treatment. Taken together, these results indicate that YTX has a clear cytotoxic effect for the MC tumour line, while primary MCs were highly resistant to YTX induced toxicity.

**Fig 3:**
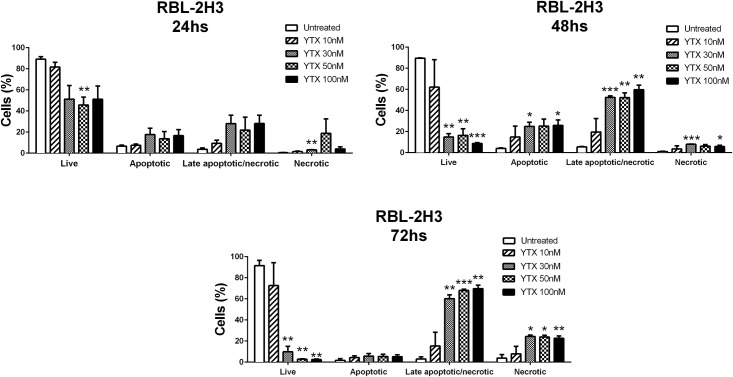
YTX treatment induced apoptosis and necrosis in the RBL-2H3 cell line. RBL-2H3 cells were either left untreated or were treated for indicated times with 10, 30, 50 and 100nM YTX. YTX solvent controls were performed and no effects were observed. Cells were then analysed for Annexin V-FITC and PI staining by flow cytometry and the percentage of live cells (Annexin V-FITC -/ PI -), early apoptotic (Annexin V-FITC +/PI -) and late apoptotic or necrotic cells (Annexin V-FITC +/PI +) was determined. Data are the mean ± SEM of three experiments. Significant differences between untreated and YTX-treated cells: (*) p≤0.05, (**) p≤0.01 and (***) p≤0.001.

The pathway by which YTX induced cell death in RBL-2H3 cells remained unclear. We therefore used an Annexin V-FITC/PI staining assay to determine whether YTX-induced apoptosis or necrosis in these cells. The assay allowed to distinguish viable (Annexin V-FITC -/PI -), early apoptotic (Annexin V-FITC +/ PI -) and late apoptotic or necrotic cells (Annexin V-FITC +/PI +). Fig 3 shows, that upon treatment of RBL-2H3 cells with different doses of YTX the cells lost cell viability in a dose dependent manner undergoing a transition from early apoptosis after 24h to late apoptotic cells, which become highly prominent at the later time points of treatment. Some necrotic cells appeared at early time points and they significantly increased before 72h. The apoptosis induction is clearly dose dependent becoming rapidly apparent at 30nM, while the lower dose of 10nM only started to show some apoptotic effects at 72h, albeit this was not significant. Taken together these results indicate that YTX induces cell death by apoptosis.

**Fig 4:**
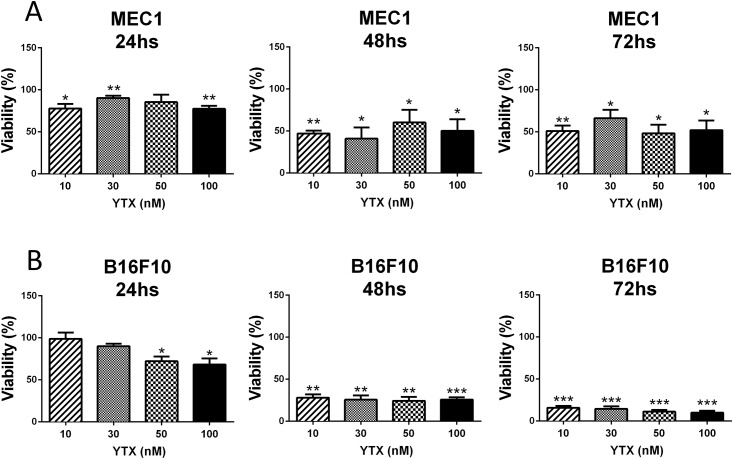
Effect of YTX on MEC1 and B16F10 cell line viability. The MEC1 and B16F10 cell lines were incubated with 10, 30, 50 and 100nM YTX for the indicated times and cell viability was assessed by the MTT assay at the indicated time points. Corresponding controls with YTX solvent were performed and cell viability was arbitrarily set to 100%. Of note, solvent did not significantly affect cell viability as compared to non-treated cells even at the highest concentration of vehicle. Data are the mean ± SEM of three experiments. Significant differences between untreated and YTX-treated cells: (*) p≤0.05, (**) p≤0.01 and (***) p≤0.001.

**Fig 5:**
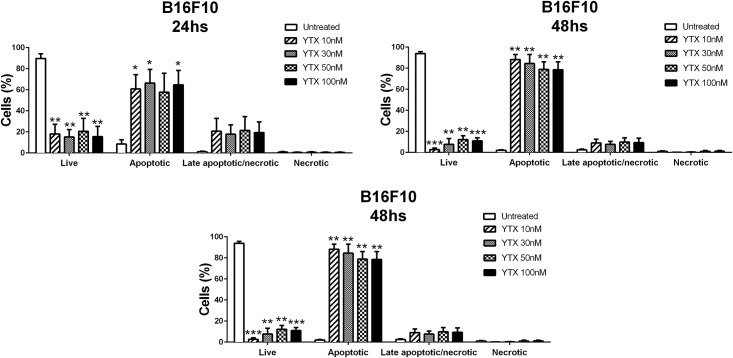
YTX treatment induced apoptosis in B16F10 cells. B16F10 cells were either left untreated or were treated for indicated times with 10, 30, 50 and 100nM YTX. YTX solvent controls were performed and no effects were observed. Cells were then analyzed for Annexin-V and PI staining by flow cytometry and the percentage of live cells (Annexin V-FITC -/PI -), early apoptotic (Annexin V-FITC +/PI -) and late apoptotic or necrotic cells (Annexin V-FITC +/PI +) was determined. Mean ± SEM of three experiments. Significant differences between untreated and YTX-treated cells: (*) p≤0.05, (**) p≤0.01 and (***) p≤0.001.

The observed results corroborate previous works confirming the anti-tumour activity of YTX [14, 15]. In order to study the most suitable cell line for the *in vivo* experiment we selected two different cell lines, MEC1 and B16F10, both of which have been used as a tumour *in vivo* mouse model in our laboratory. MEC1 is a human cell line obtained from a patient with B-chronic lymphocytic leukemia (B-CLL) in prolymphocytoid transformation [36], whereas B16F10 is a mouse melanoma cell line [27]. The cytotoxic effect of YTX treatment was studied in both cell lines using the same kinetics as above for RBL-2H3 cells. Fig 4A shows, that YTX induces a significant decrease in cell viability at 24h of treatment at 10, 30 and 100nM doses, in MEC1 cells. The cytotoxic effect of YTX was even stronger after 48h causing a significant decrease in cell viability (around 50%) at all YTX concentrations but cytotoxicity did not decrease further at 72h of incubation with YTX. For B16F10 cells the results show that YTX caused a progressive decrease in cell viability with essentially no remaining viable cells after 72h of incubation with YTX (Fig 4B). This effect was observed at all treatment doses studied. Even the smallest dose decreased viability by up to 90%. These results were confirmed when cells were stained with Annexin V-FITC and PI (Fig 5). Results showed that already after 24h a significant proportion exhibited early signs of apoptosis and that this proportion increased significantly at later time points.

**Fig 6:**
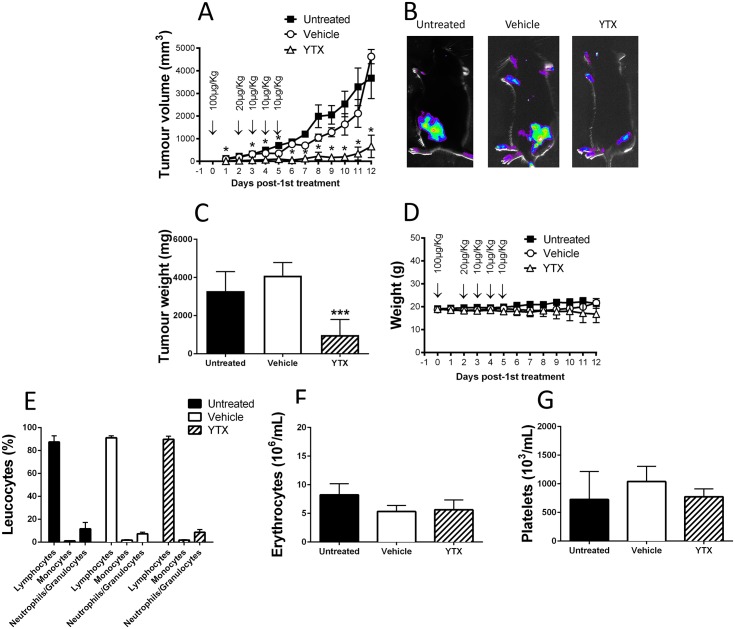
YTX treatment decreases tumour development in the B16F10 melanoma mouse model. (A) Mice were injected subcutaneous B16F10 melanoma cells and tumours were allowed to develop until they reached a volume of 50 mm^3^ achieved between days 5 and 10. Mice were then either left untreated or were treated with the indicated concentrations of YTX or vehicle, which was injected subcutaneous right next to the tumour. (B) Representative glucose uptake after FX Pro Kodak image analysis of untreated, vehicle and YTX-treated mice at the day of sacrifice. (C) Corresponding resected tumour weight of animals after sacrifice at day 12 post-1st treatment. (D) Corresponding weight of untreated, vehicle- and YTX-treated mice. (E-G) Results of the haematological analysis performed before mice sacrifice. Data are expressed as mean ± SEM. Significant differences between untreated and YTX: (*) p≤0.05 and (***) p≤0.001.

The above results indicate that YTX induces a stronger cytotoxicity towards B16F10 cells as compared to the MEC1 cell line. We therefore selected the B16F10 mouse tumour model to test the YTX anti-tumour effect *in vivo*. To this end B16F10 cells were injected subcutaneously into the flanks of the mice and allowed to grow until appearance of a small palpable tumour bleb with a size of about 50 mm^3^. Tumour treatment was started with an initial injection at high dose (100μg/Kg) in immediate tumour vicinity followed by 4 injections at lower doses at days 2, 3, 4 and 5. Fig 6A and 6B show that YTX treatment caused a clear decrease in tumour volume. This decrease was significant reducing the volume of the tumour by more than 12 times at day 10. Moreover, tumour volume did not differ between control and vehicle treated mice. Results obtained for the tumour weight after sacrifice corroborate the data observed *in vivo* with a significant decrease in tumour weight, whereas the treatment with vehicle does not have any effect (Fig 6C). With regard to the weight of the animals, no effect was observed neither after YTX nor vehicle treatment (Fig 6D) suggesting that such treatment was not particularly toxic for the mice. Furthermore, an analysis of haematological parameters was performed before sacrifice. Results obtained showed that YTX does not alter neither the percentage of white blood cells (lymphocytes, monocytes and neutrophils/granulocytes) (Fig 6E), nor erythrocytes (Fig 6F), nor platelets (Fig 6G). Supporting again the low toxicity of YTX when used for such treatment.

## Discussion

The interest in medical applications for algal toxins and in particular of YTX due to its low oral toxicity has grown in recent years. One of the first potential roles discovered was the anti-allergic role of this compound [37]. This could relate to the capacity of YTX to activate cellular phosphodiesterases (PDEs known to play a role in the activation of basophils and MCs) [17, 38, 39]. As no systematic evaluation on the capacity of YTX on MC degranulation has been reported we studied the YTX effect on MCs degranulation using primary BMMCs and the RBL-2H3 cell line. Our results demonstrate that although YTX causes a significant decrease of β-hexosaminidase release, this effect appears minor reaching a maximum of 25% inhibition in BMMCs and RBL-2H3 cells being observed in the RBL-2H3 MC line only at optimal antigen doses.

Furthermore, the present paper also demonstrated that YTX is a secondary metabolite showing an important cytotoxic effect towards the RBL-2H3 cell line at low doses in the 30 to 100nM range. Interestingly non-tumoural MCs show an extraordinary resistance as no important cytotoxic effects were observed at doses of up to 50 nM with some cytotoxicity becoming apparent at 100nM, but only after long-term incubation (72h). In addition to RBL-2H3 cells YTX exhibited also a high cytotoxic activity against MEC1 and B16F10 cell lines confirming several other studies including a National Cancer Institute human tumour cell line screen, which showed high toxicity to many tumour cell lines in the nanomolar range [18]. In particular, melanoma cell lines were the most sensitive to YTX, followed by lung, colon, leukemia, mammary, ovarian, central nervous system and renal cell lines [4, 18]. On the other hand, low toxicity was generally observed in primary cells. Thus, previous studies in fresh human lymphocytes and K-562 lymphoma cells showed that they were, respectively, resistant and sensitive to YTX [14, 15]. Differential effects of YTX also showed that it does not induce cell death features in a lymphoblastoid cell line, whereas it induced apoptosis in the lymphocytic cell line K-562 [40].

Three different cell death mechanisms can be induced by YTX. The apoptotic pathway including caspase 3 activation was first described in the BE(2)-M17 neuroblastoma cell line [13], whereas paraptosis (BC3H1 myoblast cells) [41] and autophagy (human glioma and K-562 cells) were more recently described [15, 41, 42]. Previous work also described YTX apoptosis induction in K-562 cell line (other by activating caspase 3 and 8 (extrinsic apoptotic pathway hallmark) and decreasing anti-apoptotic Bcl-2 protein levels [15]. In our present manuscript the apoptotic cell death pathway was found to be the major cause of death in RBL-2H3 or B16F10 cell lines. Indeed, YTX rapidly induces exposure of phosphatidylserine as one of the early signs of apoptosis ending in a late apoptotic stage characterized by the uptake of PI in RBL-2H3 cell line. These data suggest, while YTX can induce multiple cell death pathways, including apoptosis and the recently described paraptosis and autophagy, the apoptosis pathway seems to be the primary pathway in RBL2H3 and B16F10 cells.

Among three cell lines tested in the present study, B16F10 was shown to exhibit the highest sensitivity to YTX, followed by RBL-2H3 and MEC1. The B16F10 is a very aggressive murine melanoma cell line [43] and its high YTX sensitivity is in accordance with the results obtained in a previous wide scale screening, which showed high sensitivity of human melanoma cell lines to the compound [4]. Malignant melanoma is the sixth most common cancer in the US [27] calling for novel drugs/therapies to effectively treat melanoma patients. Cutaneous melanomas are generated from skin melanocytes and albeit recent progress has been made through the use of checkpoint inhibitors, the effectiveness of the treatment is highly variable and depends on the patient [27, 44–46]. Based on the significant *in vitro* efficacy of YTX in inducing cell death of B16F10 melanoma cells and the fact that YTX has never been evaluated as an anti-cancer drug *in vivo* we examined its efficacy *in vivo* in the B16F10 mouse melanoma model. Our results showed that local subcutaneous administration of YTX in the close vicinity to established tumour bleb resulted in a marked reduction in melanoma growth. One high dose bolus injection of 100μg/Kg followed by 4 additional low dose injections (20 and 10μg/Kg) were sufficient to reduce by 82% the B16F10 tumour melanoma volume after 12 days without affecting animal weight and haematological parameters. These results are encouraging and can be compared with other local therapeutic strategies tested in B16F10 melanoma murine model. Firstly, YTX *in vitro* cytotoxicity in B16F10 cells is 1000-fold more potent than the observed with the H-15 cyclic pentapeptide [47]. Another comparison includes the COOH-Terminal Peptide of Platelet Factor-4 Variant (CXCL4L1/PF-4var^47-70^), which when injected intra-tumourally was also described as a successful therapy in B16F10 melanoma growth *in vivo* [48]. Tumour growth inhibition due to CXCL4L1/PF-4var^47-70^ after 16 days treatment was about 60% but treatment started already at the time of inoculation with tumour cells. YTX treatment seems equivalent to therapeutic effects achieved with subcutaneous vaccination of a viral cocktail expressing a C-terminal peptide of human telomerase transcriptase, an enzyme expressed in more than 85% of human tumour cells but rarely in normal cells [33]. Therefore, the present work demonstrates, for first time, the activity of YTX as an anticancer compound *in vivo* making it a compound with a great interest in cancer therapy.

## Abbreviations

DNP: dinitrophenyl
MC: mast cell
RBL: rat basophilic leukemia
YTX: yessotoxin
